# Neonatal Disease Prediction Using Machine Learning Techniques

**DOI:** 10.1155/2023/3567194

**Published:** 2023-02-23

**Authors:** Yohanes Gutema Robi, Tilahun Melak Sitote

**Affiliations:** ^1^Department of Information Systems, College of Computing and Informatics, Haromaya University, P.O. Box 138, Dire Dawa, Ethiopia; ^2^Department of Computer Science and Engineering (CSE), School of Electrical Engineering and Computing, Adama Science and Technology University, P.O. Box 1888, Adama, Ethiopia

## Abstract

Neonatal diseases are among the main causes of morbidity and a significant contributor to underfive mortality in the world. There is an increase in understanding of the pathophysiology of the diseases and the implementation of different strategies to minimize their burden. However, improvements in outcomes are not adequate. Limited success is due to different factors, including the similarity of symptoms, which can lead to misdiagnosis, and the inability to detect early for timely intervention. In resource-limited countries like Ethiopia, the challenge is more severe. Low access to diagnosis and treatment due to the inadequacy of neonatal health professionals is one of the shortcomings. Due to the shortage of medical facilities, many neonatal health professionals are forced to decide the type of disease only based on interviews. They may not have a complete picture of all variables that have a contributing effect on neonatal disease from the interview. This can make the diagnosis inconclusive and may lead to a misdiagnosis. Machine learning has great potential for early prediction if relevant historical data is available. We have applied a classification stacking model for the following four main neonatal diseases: sepsis, birth asphyxia, necrotizing enter colitis (NEC), and respiratory distress syndrome. These diseases account for 75% of neonatal deaths. The dataset has been obtained from the Asella Comprehensive Hospital. It has been collected between 2018 and 2021. The developed stacking model was compared to three related machine-learning models XGBoost (XGB), Random Forest (RF), and Support Vector Machine (SVM). The proposed stacking model outperformed the other models, with an accuracy of 97.04%. We believe that this will contribute to the early detection and accurate diagnosis of neonatal diseases, especially for resource-limited health facilities.

## 1. Introduction

The neonatal period is a critical time in human life when a newborn baby has to adapt to a new environment and complete several physiological adjustments that are essential for life [1]. Neonatal mortality is a significant contributor to underfive mortality [1]. According to estimates for 2018, more than 2.4 million children died before their second month of life [2]. The neonatal mortality rate shows differences between regions and nations. One-third of the world's neonatal deaths are from sub-Saharan Africa, with about 34 deaths per 1000 live births. The risk of neonatal death is approximately 55 times higher in the country with the highest mortality rate than in the country with the lowest mortality rate [3]. The neonatal mortality rate in Ethiopia is about 30 per 1000 live births [4]. The region is falling short of achieving Sustainable Development Goal 3 (SDG-3) [5].

The leading neonatal diseases are sepsis, respiratory distress syndrome, birth asphyxia, and necrotizing enter colitis accounting for 26%, 23%, 19%, and 7%, respectively [6–8]. In Ethiopia, the most common diseases leading to neonatal death are sepsis, birth asphyxia, necrotizing enter colitis (NEC), and respiratory distress syndrome (RDS) [4]. Contributing factors for neonatal death include shortages of neonatologists and pediatricians, the inadequacy of diagnostic tools, diagnostic delay, and lack of quality care and treatments for neonatal conditions [9]. Some neonatal diseases have similar symptoms, which often result in the inappropriate use of antibiotics, which increases the risk of the development of antimicrobial resistance. For instance, neonatal sepsis is very similar to diseases such as perinatal asphyxia and necrotizing enter colitis which makes it difficult to accurately diagnose and treat. In resource-limited countries like Ethiopia, neonatal diseases exert a heavy burden on families, society, and the health system. There are preventive and curative strategies to mitigate the impact. But there are limited improvements in the outcomes. Preventive approaches focus on maternal health before birth, such as maternal immunization and efforts to guarantee a healthy pregnancy [10, 11]. With respect to curative approaches, there are limited diagnostic tools, and the results of diagnostics take longer. The delay in results often leads to a neonate's condition rapidly deteriorating [12]. It has serious repercussions including chronic lung disease, neurodevelopmental abnormality, and long-term impairment that necessitate continuous hospitalization [13–16]. There are also significant increases in expenses and burdens for both survivors and caregivers. Hence, early identification of neonatal disease with appropriate antibiotic therapy can be effective in reducing neonatal death, reducing cost, and lowering antibiotic resistance in the community [17]. Detection of diseases at an early stage with minimum cost is an area of interest to many researchers [18]. Previous studies have shown the effectiveness of machine learning techniques in early recognition for timely preemptive clinical intervention [19]. There have been successful applications of single classifiers, ensemble techniques, stacking, and hybrid machine learning methods [20]. Late-onset sepsis (LOS) is one of the major contributors to morbidity and mortality in neonates. Early detection of LOS is critical to reduce related illnesses and death. Machine learning techniques have been used effectively for the early recognition of LOS [21]. By identifying disease beginning before it becomes clinically evident and starting antibiotic medication on time, it may be possible to avert negative outcomes in newborns.

In this study, we used a stacking machine learning model to classify the following four major neonatal diseases: sepsis, birth asphyxia, necrotizing enter colitis (NEC), and respiratory distress syndrome, which account for 75% of neonatal deaths. The dataset was obtained from the Asella Comprehensive Hospital. It has been collected between 2018 and 2021. Comparisons have been made between the developed stacking model and selected machine learning models such as XGBoost, Random Forest (RF), and Support Vector Machine (SVM) with and without feature selection.

The paper's remaining part has been organized into four sections. In Section 2, related works on neonatal disease prediction have been discussed.  Section 3 contains materials and methods. The following topics have been covered: dataset, preprocessing, proposed machine learning model, and evaluation. In Section 4, experiments, results, discussions, and evaluations of the proposed method were incorporated. Lastly, the conclusion that highlights the major findings and inferences has been incorporated in Section 5.

## 2. Related Works

Machine learning approaches have a lot of potential considering high-risk neonates receive intensive care that is getting more and more complicated. It has been used in numerous studies to forecast neonatal illnesses and mortality. Selected related studies on neonatal disease prediction have been discussed.

Supervised machine learning techniques have been used for the diagnosis of neonatal diseases, and some of them have been explored for their comprehensive application to analyze neonatal data by Shirwaikar et al., [22]. They have critically analyzed and discussed the methods and performance metrics of supervised techniques used on neonatal data to suggest ways to improve performance. From their review, the ensemble technique has better predictive power than SVM, neural networks, and decision trees.

Sheikhtaheri et al. applied machine learning techniques to improve the performance of prediction of neonatal mortality and its risk [23]. The dataset was collected from Iran in two phases. The factors that lead to infant death, including diseases, were initially identified before training, testing, and evaluating the effectiveness of several algorithms, such as ANN, RF, CHART, SVM, and ensembles. SVM had the best accuracy of 94%.

Using a BP learning algorithm, Chowdhury et al., trained a multilayer perception to identify a design pattern for the prediction of neonatal illnesses. They compared their approach with different algorithms that have been previously used for the prediction of neonatal diseases such as conjugate gradient descent and quick propagation. The proposed model used 94 cases of different symptoms and signs as a parameter to test the model and obtained 75% accuracy [24].

Safdari et al. developed an expert system with fuzzy logic that predicts the risk of neonatal death. To gain knowledge, they created questionnaires and distributed them to neonatologists [25]. Then, they combined computational and fuzzy models based on an inference system for the prediction of neonatal death risk. They used MATLAB for model building and C# for the graphical user interface (GUI). The model has a 90% accuracy.

Shirwaikar et al. applied machine learning techniques to predict episodes of apnea in preterm neonates. They have only considered neonates who are not older than one week. The 229 neonates admitted to the neonatal intensive care unit (NICU) make up the dataset. SVM, RF, and decision trees have been used to predict apnea episodes in neonates. RF outperforms the other machine learning models with an accuracy of 88% [26]. They have developed a machine learning-based automated solution to predict apnea in neonates.

Mani et al. have developed machine-learning models to predict LOS using secondary data from electronic medical records (EMR) [17]. Comparisons have been made between predictions made by models resulting from machine learning algorithms and the sepsis treatments administered by physicians. The outcome was impressive, with eight out of nine machine learning algorithms tested have outperformed physicians in terms of treatment sensitivity, and all nine machine learning algorithms are superior in terms of specificity.

There are studies in Ethiopia to predict neonatal diseases and mortality. Bitew et al. showed the risk of underfive mortality in Ethiopia using RF, LR, and KNN [27]. They tried to identify important sociodemographic determinants using the 2016 EDHS dataset. RF has the highest accuracy of 67.2%. Different regions of Ethiopia have different underfive mortality rates. The summary of selected related works is shown in Table 1.

## 3. Materials and Methods

**Figure 1:**
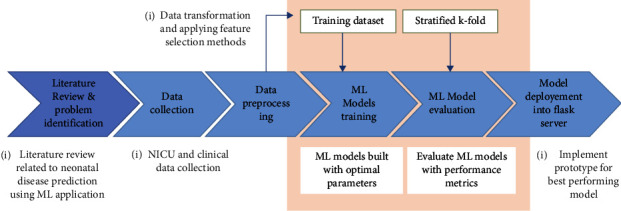
Workflow.

In this study, four high-burden neonatal diseases such as sepsis, birth asphyxia, necrotizing enter colitis (NEC), and respiratory distress syndrome have been classified using a stacked machine learning approach. The dataset was obtained from Asella Compressive Hospital.  Figure 1 shows the overall workflow.

**Figure 2:**
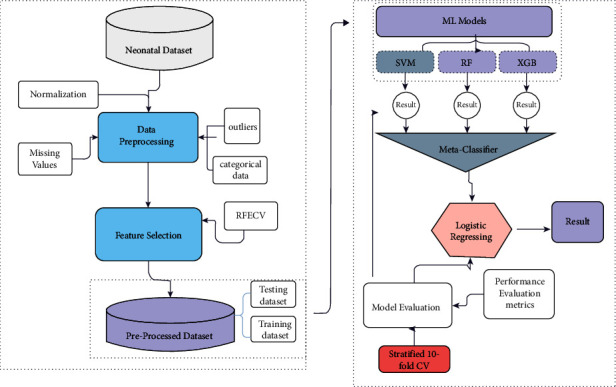
Neonatal disease prediction architecture.

The proposed architecture has been shown in Figure 2. Steps starting from collecting relevant data to evaluation have been followed. The dataset undergoes preprocessing including cleaning, handling missing values, and transforming the data. Recursive feature elimination with cross-validation has been chosen as an appropriate feature selection technique to identify relevant features. Then, preprocessed data was fed into SVM, RF, and XGB. The results of three selected models have been combined to form stacking. The models' performances were evaluated using stratified k-fold cross-validation (*k* = 10) with and without feature selection methods. These steps and techniques have been discussed in the following sections:

### 3.1. Data Collection

Data used for this research was obtained from neonatal patient cards' of patients admitted to the NICU of Asella Comprehensive Hospital, Asella, Oromia, Ethiopia, during the period of 2018 to 2021. The hospital keeps the record of each patient in a manual format. The primary task in the data collection was to carefully encode each instance into a soft copy. It was compiled from neonatal disease discharge summaries and examination cards. The three-year dataset has 2298 instances with 20 features. The registered dataset includes admission information, delivery information, symptoms, laboratory results, and X-ray results. A description of the features of the dataset is shown in Table 2. Experts working in the NICU reviewed the patient history dataset. To enhance our understanding of the situation and features, we conducted interviews with pediatricians. We have also assessed different local and global literature on neonatal disease.

### 3.2. Preprocessing

**Figure 3:**
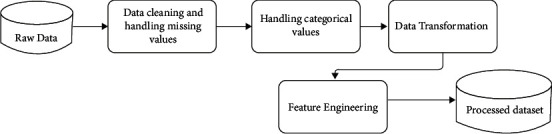
Preprocessing.

The dataset of the study contains incomplete, noisy, inconsistent, inaccurate, and irrelevant values. Preprocessing has been carried out before modeling, as shown in Figure 3.

#### 3.2.1. Cleaning Data and Missing Values Handling

Missing values can be handled in several ways, including by dropping them if they have an insignificant impact on individual instances, replacing them with a global constant, imputation, and predicting missed values. In the dataset, 12 features contain missing values, as shown in Table 3. The missing values were filled up via imputation using mean values for categorical features and mode values for numeric features.

#### 3.2.2. Handling Imbalanced Data and Feature Scaling

The dataset has a slight class imbalance. This has been handled by setting the class weight of the hyperparameter setting. Standardized scalar has been used for feature scaling in this study.

Standardize scalar

(1) $$\begin{array}{c} X^{\prime }=\frac{x-u}{s}\text{,} \end{array}$$

where *X* is the score of a sample, *u* is the training sample mean and *s* is the standard deviation.

#### 3.2.3. Selection of Features

One of the preprocessing steps is identifying the feature set that is relevant to generate the best possible result with a feasible computational cost. It is the process of deciding which feature set, typically from a large number of input features, is the most important because not all features will necessarily be useful. Hence, the primary goal of feature selection is to choose an essential set of features to reduce the computational cost without compromising the performance of the model. Clinical datasets frequently use a filter, wrapper, and embedded feature selection approaches [28–31]. By evaluating the correlation between features and the target feature, the most important features are chosen using the filter approach. It is independent of the machine-learning algorithm. Another popular feature selection method is the wrapper method, which selects a set of features as a search problem in which several combinations are generated, estimated, and compared with one another. Univariate, recursive feature elimination, and sequential forward selection are better methods. Effective techniques for selecting features include recursive feature elimination (RFE). It is efficient at picking out the most essential features. Hence, recursive feature elimination with cross-validation (RFECV) has been chosen in this study.

### 3.3. Modeling

**Figure 4:**
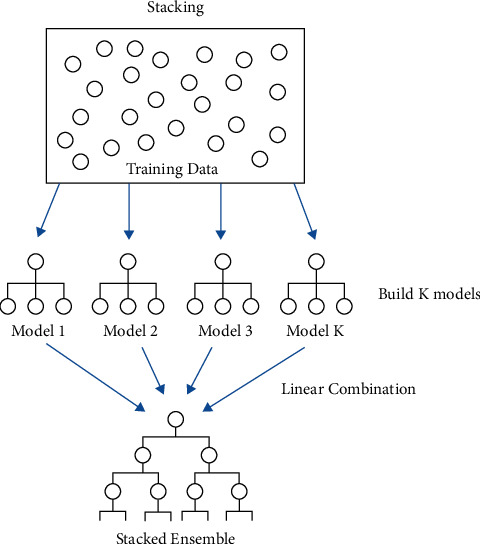
Stacking classifier.

Instead of individual learners, we used the stacking approach, which is one of the most successful approaches to classification and regression problems. If appropriately applied, multilevel stacking generates more precise results than individual models. In stacking, individual model predictions from the prior level are used as input for models in the subsequent level, like meta-learner [32]. It combines multiple classifiers or models *M*1, *M*2,…, *M*n on a single dataset S [33]. *S* consists of examples *si* = (*xi*, *yi*), i.e., pairs of feature vectors (*xi*) and their classifications (*yi*). It started with the generation of base-level classifiers *C*1,…, *Cn*, where *Ci* = *Mi* (*s*). Second, the output of the base-level classifiers is used as input by the meta-level learner. Cross-validation has been applied to create a training set for the meta-level classifier. The procedure continues, as shown in Figure 4.

**Figure 5:**
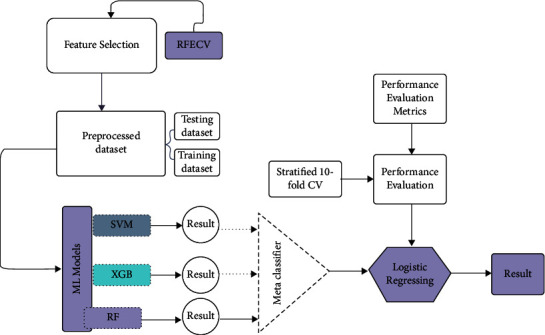
Model building flow diagram.

Three base-level learners; SVM, RF, and XGB, have been combined for stacking with and without feature selection. The model-building workflow has shown in Figure 5, and the base-level learners have been discussed in the following subsections:

#### 3.3.1. Support Vector Machine (SVM)

**Figure 6:**
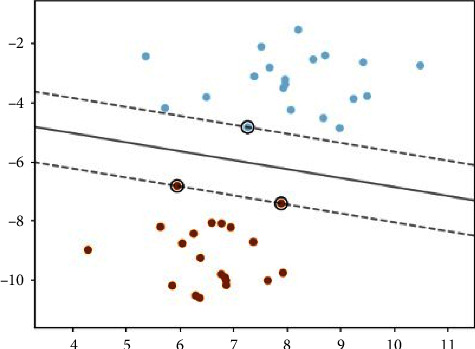
Decision function for the linearly separable problem.

SVM is a collection of similar classification and regression learning methods. It can be linear, multiple, or nonprobabilistic. The primary goal is to find the best possible boundary between classes. In order to classify data, SVM creates a hyperplane or set of hyperplanes in a high-dimensional space, as shown in Figure 6. The data points on the opposite side of the hyperplane belong to different classes. The longer the hyperplane's distance from the closest training data points, the better the separation for classification. Hence, the longer the margin, the smaller the classifier's error. In this study, we used the Support Vector Machine (SVM) and machine learning classifier's One-Vs-Rest (OVR) strategy. We used the OVR with SVM since it is widely used for multiclass classifications.

#### 3.3.2. Random Forest (RF)

It is an ensemble of classifiers that can solve classification and regression problems and is often composed of a decision tree. This technique generates a forest of several decision trees at random. The result is more precise when there are more trees in the forest. The way RF operates is to first select *K* randomly chosen data points from the training sample. It then creates decision trees associated with the selected data points. It then repeats steps 1 and 2 after selecting the number *N* for the intended decision trees to be built. It also identifies the predictions made by each decision tree and assigns the new data instances to the category with the most votes.

#### 3.3.3. XGBoost (XGB)

XGBoost is an extended version of gradient-boosting decision trees designed for the speed and performance of machine learning. XGBoost is used for both classification and regression tasks. Important features of XGBoost are as follows:

- Parallelization: implemented on multiple CPU cores to train
- Regularization: XGBoost uses different regularizations to avoid overfitting
- Nonlinearity: the ability to generate nonlinear data.
- Cross-validation: built-in
- Scalability

#### 3.3.4. Hyperparameter Tuning

Hyperparameter tuning is a method of selecting a group of hyperparameters to optimize performance. The tuning can be carried out manually or automatically. Manually, different sets of hyperparameters are selected and tested. This is tiresome and may not be feasible when we have a large number of hyperparameters to try. But with automatic approaches, an optimization algorithm is used to select the optimal set of hyperparameters. In this study, we have used the automatic method. The two most popular algorithms are grid search and random search. A grid search is a common technique for hyperparameter optimization that conducts a complete search on a predetermined subset of the algorithm's hyperparameter space. Candidates are generated during training using a particular grid of parameter values. High-dimensional spaces are problematic for this approach. Grid searches are inferior to random searches, especially when only a small number of hyperparameters affect the performance of the machine learning algorithm. Hence, a random search has been used for this study.

### 3.4. Evaluation

Evaluation techniques have been used to evaluate the performance of the proposed model. The performance evaluation method may be holdout or cross-validation. By testing a model on data other than the ones used to train it, holdout evaluation attempts to provide an objective assessment of learning performance. A large dataset is divided into two subsets at random using this basic strategy, such as training and testing sets. The machine learning models are trained with the training dataset. The models' performance is then tested using an unseen testing dataset. *K*-foldcross-validation is the technique used for evaluating a model's performance on an unseen test dataset. The stratified form of the *k*-fold cross-validation enforces matching the class distribution in each split with the entire training dataset. Due to the availability of a slightly imbalanced class distribution, we believe that stratified *k*-fold cross-validation is appropriate. Hence, it has been used in this study.

The performance of selected models has been evaluated using various performance evaluation metrics, including precision, recall, accuracy, and *f*1-score. When classification is conducted, four different kinds of results could be found as follows:

- True positive (TP) is a result when the model correctly predicts positive class instances, i.e., the predicted positive value is the same as the actual positive value
- True negative (TN) is a result when the model correctly predicts negative class instances, i.e., the predicted negative class value is the same as the actual negative class value
- False positive (FP) is a result when the model wrongly predicts as positive class value when the actual value is negative
- False negative (FN) is a result when the model wrongly predicts as negative class value when the actual value is positive

Accuracy is a widely used evaluation metric for classification models. It is a percentage of correctly classified values as shown in the following equation:

(2) $$\begin{array}{c} Accuracy=\frac{TP+TN}{TP+TN+FP+FN}\text{.} \end{array}$$

Precision is the ratio of true positive to the sum of true positive and false positive values as shown in the following equation:

(3) $$\begin{array}{c} Precision=\frac{TP}{TP+FP}\text{.} \end{array}$$

Recall is the ratio of true positives to the number of all relevant samples as shown in the following equation:

(4) $$\begin{array}{c} Recall=\frac{TP}{TP+FN}\text{.} \end{array}$$

*F*1-score is calculated with the harmonic mean of precision and recall as shown in the following equation:

(5) $$\begin{array}{c} F1-Score=2*\frac{Precision*Recall}{Precision*Recall}\text{.} \end{array}$$

Table 4 shows the confusion matrix.

## 4. Results and Discussion

In this section, dataset exploration, feature selection, modeling, and evaluation have been discussed. The results of selected models and a newly developed stacking model were compared. The best-performing model has been deployed using a Flask server. A comparative discussion of the results with those of previous studies has also been made.

### 4.1. The Dataset Exploration

**Figure 7:**
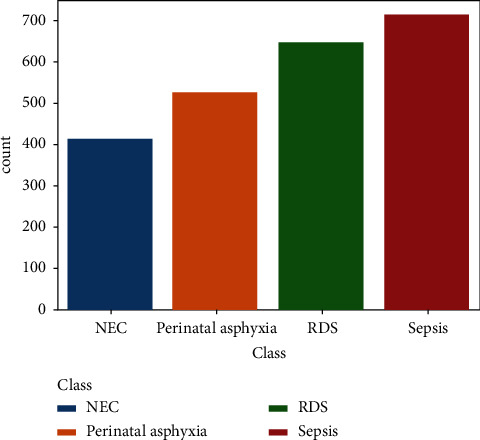
Distribution of four neonatal diseases.

The total size of the dataset is 2298, with 20 features including the target class. Four dominant neonatal diseases considered in the study are sepsis, respiratory distress syndrome (RDS), necrotizing enterocolitis (NEC), and parental asphyxia (PA). Their distribution has been shown in Figure 7, which is 711 instances of sepsis, 648 instances of respiratory distress syndrome (RDS), 527 instances of parental asphyxia (PA), and 412 instances of necrotizing enterocolitis (NEC). There is a slight class imbalance.

**Figure 8:**
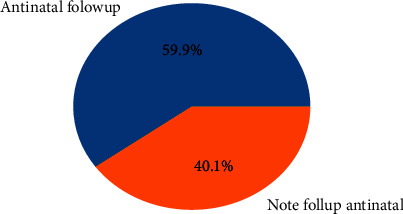
Antenatal follow-up.

**Figure 9:**
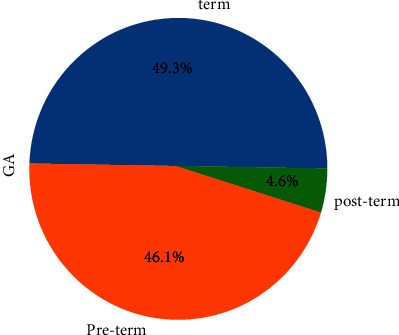
Term birth status.

As shown in Figure 8, 59.9% of women follow up on antenatal care during their pregnancy. As shown in Figure 9, 49.3% of neonates were born term, 4.6% were born preterm, and 46.1% were born post-term.

### 4.2. Feature Relevance

**Figure 10:**
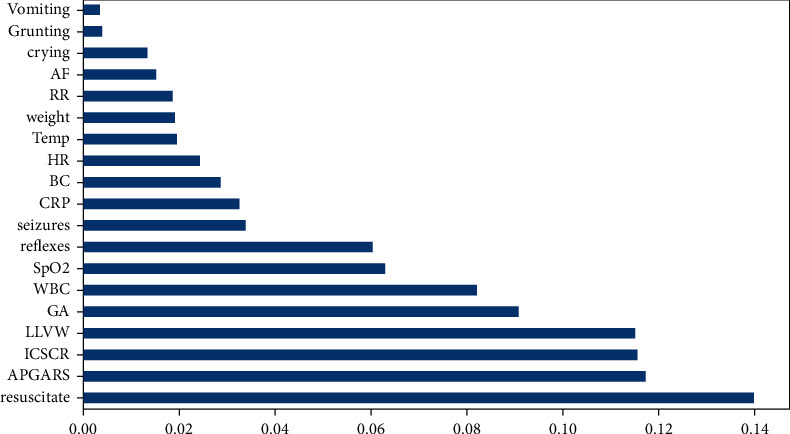
Feature importance.

The ranking of features based on their relevance has been shown in Figure 10. Feature selection methods have been applied in order to select relevant feature sets for the better predictive performance of classifiers with an acceptable computational cost. Recursive feature elimination with cross-validation (RFECV) was used in the training of the SVM, RF, XGB, and stacking ensemble models. As a result, 12 features were selected.

Models were built on multiclass datasets with and without feature selection techniques. Stratified 10-fold cross-validation has been used along with other evaluation methods, as previously discussed. Stacking, SVM, RF, and XGB performance have been discussed using the original features of the neonatal disease dataset without any feature selection.

**Figure 11:**
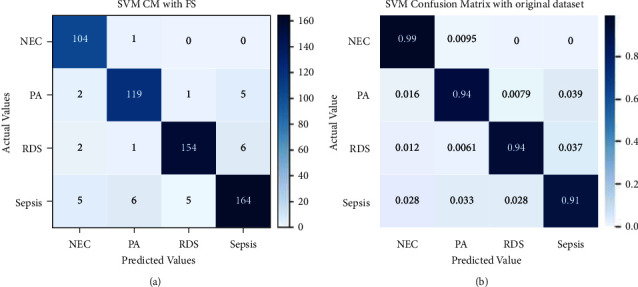
Confusion matrix for SVM without (a) and with normalization (b).

The performance of SVM has been shown in the confusion matrix in Figure 11. 104 instances of NEC out of 105, 119 instances of PA out of 127, 154 instances of RDS out of 163, and 164 instances of sepsis out of 180 have been correctly classified. It wrongly classified 1 instance of NEC as PA, 2 instances of PA as NEC, 1 instance of PA as RDS, and 5 instances of PA. Similarly, the other wrongly classified can also be seen from the figure. The normalized confusion matrix is displayed in Figure 11(b), and it is identical to Figure 11(a) except that it displays instances that were correctly identified as decimal.

**Figure 12:**
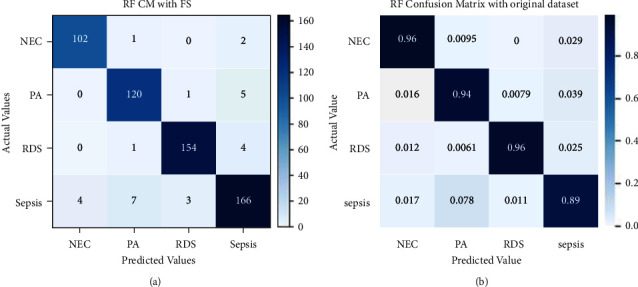
Confusion matrix for RF without (a) and with normalization (b).

Figure 12 shows a confusion matrix used to assess Random Forest's performance. It correctly classified 102 instances of NEC out of 105, 120 instances of PA out of 127, 158 instances of RDS out of 163, and 166 instances of sepsis out of 180. RF only misclassified 1 instance of NEC as PA and 2 instances of NEC as sepsis. Similarly, the other misclassifications can be seen in the figure. Instances that have been correctly classified in decimals have been shown in the normalized confusion matrix.

**Figure 13:**
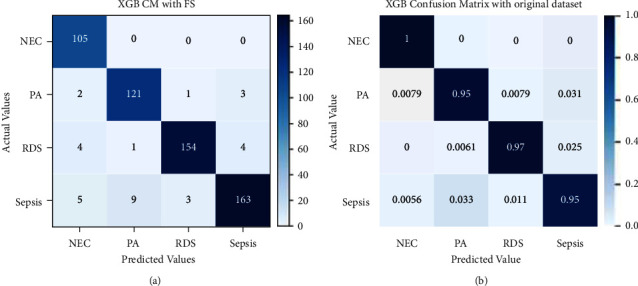
Confusion matrix for XGB without (a) and with normalization (b).

The other classifier that has been used is XGB and its performance is shown in Figure 13. It correctly classified instances of 105 out of 105, 121 out of 127, 154 out of 163, and 163 out of 180, as Sepsis, NEC, RDS, and PA, respectively. Misclassifications can be seen in the figure. The confusion matrix in Figure 13(b) is identical to Figure 13(a) with the exception that it has been normalized.

The evaluation results of RF, XGB, SVM, and stacking models without feature selection have been summarized in Table 5. Stacking's score is the highest in all the following four performance matrices: precision, recall, *F*1-score, and accuracy.

The next set of experiments were using RFE to choose the best feature subset with the objective of enhancing the performance of models. The evaluation results of RF, XGB, SVM, and stacking models with feature selection have been discussed in the paper.

**Figure 14:**
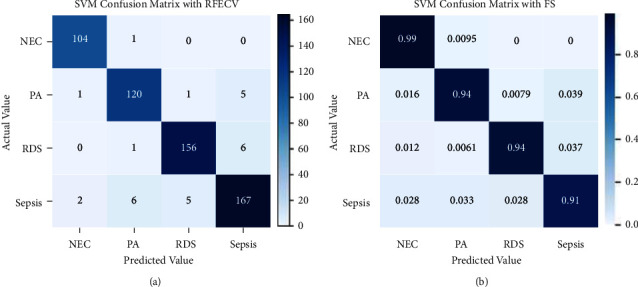
Confusion matrix for SVM with RFECV without and with normalization.

The evaluation result of SVM using recursive feature elimination with cross-validation is shown in Figure 14. 104 instances of NEC out of 105, 120 instances of PA out of 127, 156 instances of RDS out of 163, and 167 instances of sepsis out of 180 have been correctly classified. There are few wrongly classified values.  Figure 14(b) shows a normalized confusion matrix for SVM with RFECV.

**Figure 15:**
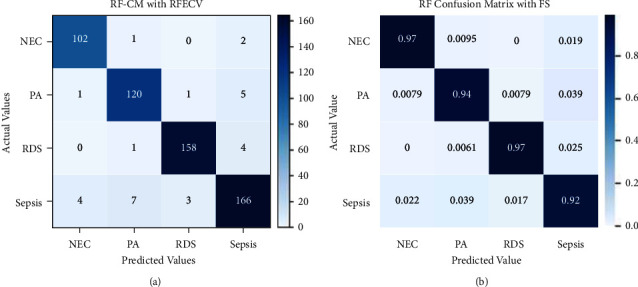
Confusion matrix for RF with RFECV without and with normalization.

The confusion matrix performance evaluation result of the Random Forest model with recursive feature elimination and cross-validation has been illustrated in Figure 15. 102 instances of NEC out of 105, 120 instances of PA out of 127, 158 instances of RDS out of 163, and 166 instances of sepsis out of 180 have been correctly classified. There are a few wrongly classified instances.  Figure 15(b) shows normalized evaluation results for RF with RFECV.

**Figure 16:**
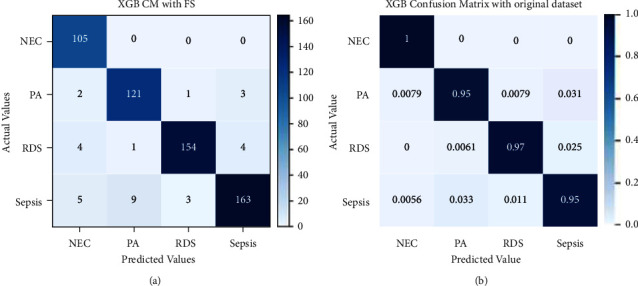
Confusion matrix for XGB with RFECV without and with normalization.

The performance evaluation results of the XGBoost model with recursive feature elimination and cross-validation have been illustrated in Figure 16. 105 instances of NEC out of 105, 121 instances of PA out of 127, 154 instances of RDS out of 163, and 163 instances of sepsis out of 180 have been correctly classified. There are few wrongly classified instances.  Figure 16(b) shows a normalized confusion matrix for XGBoost with RFECV.

**Figure 17:**
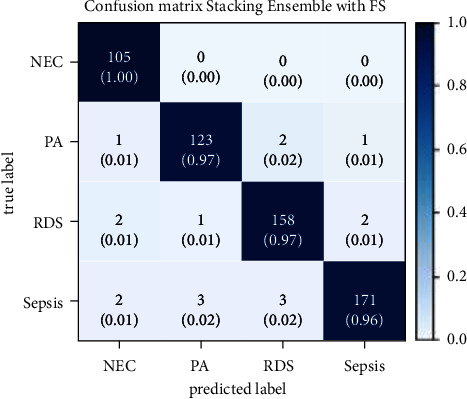
Confusion matrix for stacking with FS.

The confusion matrix of the stacking model with recursive feature elimination and cross-validation has been illustrated in Figure 17. The 105 instances of NEC out of 105, the 123 instances of PA out of 127, the 158 instances of RDS out of 163, and the 171 instances of sepsis out of 180 have been correctly classified. There are very few wrongly classified instances.

The stratified 10-fold cross-validation with recursive feature elimination evaluation result of SVM, RF, XGB, and stacking is shown in Table 6. Stacking's score is the highest in the following four performance matrices: precision, recall, *F*1-score, and accuracy. It outperformed three models in all performance matrices.

Although direct comparisons are difficult due to dataset differences, population differences, and other differences, we identified that the developed stacking model has better performance when compared to the results of previous works, as shown in Table 7.

One of the main results is the improved performance of the machine learning model by combining base models, known as stacking. Different experiments have been carried out to improve predictive performance. The APGAR score, CRP (C-reactive protein), resuscitate, LLVW (low lung volume and whiteout), ICSCR (intercostal subcostal retractions), blood cultures, SpO_2_ (oxygen saturation), GA (gestational age), WBC (white blood cells), seizures, RR (respiratory rate), weight, and grunting are the major features used to predict neonatal diseases. The stacking model outperforms three base models; Random Forest, Support Vector Machine, and XGB, with and without feature selection. Models with RFECV perform better than models with original features. The stacking model's accuracy, precision, recall, and *f*1-score are 97.04%, 97.21%, 97.38%, and 97.30%, respectively.

## 5. Conclusion

Deaths caused by neonatal diseases are a significant global contributor to underfive mortality. There are advancements to combat the challenge, including an enhanced understanding of the pathophysiology of the diseases and technological assistance for diagnosis and treatment. But the improvement is limited. The similarity of disease symptoms, which may lead to misdiagnosis, and the inability of early diagnosis for timely intervention are among the factors contributing to limited success. Neonatal disease is a major child health challenge in resource-limited countries like Ethiopia. In Ethiopia, neonatal mortality accounts for 43.3% of underfive mortality, which indicates that it has to get adequate attention and prioritization to sustain the intended progress in the reduction of child mortality. Early detection of neonatal diseases is believed to have an important contribution. In this study, the main aim was to detect and classify four major neonatal diseases (NEC, PA, RDS, and sepsis) using machine learning techniques. The data was gathered at Asella Compressive Hospital in Oromia, Ethiopia. It has 2298 instances and 20 features. Different preprocessing techniques have been applied to the dataset, including handling missing values with mean imputation, standard scaling, converting categorical features with label encoders, and class balancing. Further, recursive feature elimination with cross-validation has been applied to choose a relevant set of features. Then, modeling has been carried out using four machine learning models, such as stacking, RF, XGB, and SVM, with stratified 10-fold cross-validation. The performance evaluation showed that stacking with RFECV feature selection outperformed the other models with an accuracy of 97.04%. We believe that this will be useful for accurate diagnosis and early detection of neonatal diseases.

## Figures and Tables

**Table 1:** Summary of related works.

Title	Machine learning methods and findings	Shortcoming and pitfalls
Supervised learning techniques for analysis of neonatal data [22]	ANN, NB, SVM, and LR have been used. The highest accuracy is 89% with SVM	The dataset is small with only demographic features. There is also a high-class imbalance
Prediction of neonatal deaths in NICUs: development and validation of machine learning models [23]	ANN, RF, CHART, SVM, and ensembles have been used. The highest accuracy is 94% with SVM	The dataset is imbalanced
An artificial neural network model for neonatal disease diagnosis [24]	ANN has been used. The accuracy is 75%	The dataset contains 94 rows which is too small. Clinical features, which are very essential in predicting neonatal disease, were not used
Developing a fuzzy expert system to predict the risk of neonatal death [25]	A fuzzy model inference system has been used. The accuracy is 90%	Comparisons were not made with similar machine-learning techniques. The reason for using fuzzy was not also adequately justified
Machine learning techniques for neonatal apnea prediction [26]	DT, SVM, and RF have been used. The highest accuracy is 88% with RF	The small dataset and the selected machine-learning models were not adequately justified
Medical decision support using machine learning for early detection of late-onset neonatal sepsis [17]	SVM, NB, and its variants TAN and AODE, K-nearest neighbor, CART, RF, LR, and LBR. Machine learning models outperformed physicians	Bias may be introduced in the method of conversion of temporal variables
Machine learning approach for predicting underfive mortality determinants in Ethiopia: evidence from the 2016 Ethiopian demographic and health survey [27]	RF, LR, and KNN have been used. The highest accuracy is 67.2% with RF	Clinical features were not used and the accuracy was low

**Table 2:** Description of the neonatal disease dataset.

No	Symbol	Feature's full name	Data type	Description
1	GA	Gestational age	Nominal	It is the period between conception and delivery that has been classified as preterm, term, and post-term
2	Temp	Temperature (°C)	Numeric	It is the specific level of a newborn's body's heat or cold measured in Celsius (°C)
3	RR	Respiratory rate (bpm)	Numeric	It refers to how many breaths a newborn takes per minute
4	Reflexes	Reflexes	Nominal	It is a newly developed behavioral pattern in neonates
5	APGAR score	Appearance, pulse, grimace, activity, and respiration score	Numeric	If the score is between 6 and 0, the newborn is normal. If the score is below 6, the newborn baby needs medical attention
6	Resuscitate	Resuscitate	Nominal	Emergency procedures performed by a doctor to support newborns who are at risk
7	Seizures	Seizures	Nominal	Triggered by sudden, unusual, and excessive neuronal activity in the brain
8	HR	Heart rate (bpm)	Numeric	Is the number of times each minute that newborn heart beats which is normally between 70 and 190 bpm
9	SpO2	Oxygen saturation (pulse oximetry)	Numeric	It is the proportion of oxygen-carrying hemoglobin to nonoxygen-carrying hemoglobin in the blood that is measured in pulse oximetry
10	Crying	Crying	Nominal	
11	CRP	C-reactive protein (mg/L)	Numeric	It is the measurement of c-reactive protein in baby blood in milligrams per liter
12	WBC	White blood cells (/*μ*L)	Numeric	It is a measurement of white blood cells
13	LLVW	Low lung volumes and whiteout	Nominal	Complications are caused by changes in lungs that are not well developed. Lung whiteout is the case when the black in a lung field is replaced by white
14	Weight	Weight	Numeric	
15	ICSCR	Intercostal subcostal retractions	Nominal	It occurs when the airways are highly resistant or the lungs' compliance is low
16	Grunting	Grunting	Nominal	The sound created by the sudden closing of the glottis
17	Vomiting	Vomiting	Nominal	It is a stomach-content vomit
18	BC	Blood cultures	Nominal	It is the gold standard method for detecting blood bacteria or fungi.
19	AF	Antenatal follow-up	Nominal	Supervision of humans during pregnancy to monitor the progress of a fetus's growth
20	Diseases	Class	Nominal	Target classes containing NEC, Sepsis, RDS, and perinatal asphyxia

**Table 3:** Missing values.

Name of features	Data type	Number of missing values	Missing values in (%)
AF	Nominal	14	0.61
Grunting	Nominal	7	0.3
WBC	Numeric	12	0.52
Vomiting	Nominal	23	1
LLVW	Nominal	3	0.13
Respiratory rate (RR)	Numeric	120	5.22
Blood cultures	Nominal	53	2.31
C-reactive protein (CRP)	Nominal	25	1.1
Grunting	Nominal	49	2.13
Temperature	Numeric	22	0.96
Weight	Numeric	182	7.92
Heart rate	Numeric	295	12.84

**Table 4:** Four-class classification confusion matrix.

Predicted values					
Actual values		Sepsis	RDS	PA	NEC
	Sepsis	True sepsis	False RDS	False PA	False NEC
	RDS	False sepsis	True RDS	False PA	False NEC
	PA	False sepsis	False RDS	True PA	False NEC
	NEC	False sepsis	False RDS	False PA	True NEC

**Table 5:** SVM, RF, XGB, and stacking models performance metrics without FS.

Models	Precision (%)	Recall (%)	*F*1-score (%)	Accuracy
SVM	95.14	95.68	95.11	94.86
RF	95.8	95.70	95.70	95.78
XGB	96.01	96.13	95.79	95.91
Stacking	**96.70**	**97.0**	**96.90**	**96.69**

Bold values indicate the outperformance results of the proposed stacking classifier without feature selection compared to other models.

**Table 6:** SVM, RF, XGB, and stacking models performance results with FS.

Models	Precision (%)	Recall (%)	*F*1-score (%)	Accuracy
SVM	95.43	95.63	95.11	95.3
RF	96.66	96.98	96.67	96.43
XGB	97.02	97.16	97.11	96.65
Stacking	**97.21**	**97.38**	**97.30**	**97.04**

Bold values indicate the outperformance results of the proposed stacking classifier with feature selection compared to other models.

**Table 7:** Comparison of the proposed model with previous related works.

No	Title	Models and accuracy
1	Supervised learning techniques for analysis of neonatal data [22]	ANN, NB, SVM, and LR have been used. The highest accuracy is 89% with SVM
2	Prediction of neonatal deaths in NICUs: development and validation of machine learning models [23]	ANN, RF, CHART, SVM, and ensembles have been used. The highest accuracy is 94% with SVM
3	An artificial neural network model for neonatal disease diagnosis [24]	ANN has been used. The accuracy is 75%
4	Developing a fuzzy expert system to predict the risk of neonatal death [25]	A fuzzy model inference system has been used. The accuracy is 90%
5	Machine learning techniques for neonatal apnea prediction [26]	DT, SVM, and RF have been used. The highest accuracy is 88% with RF
6	Medical decision support using machine learning for early detection of late-onset neonatal sepsis [17]	SVM, NB, and its variants TAN and AODE, K-nearest neighbor, CART, RF, LR, and LBR. Machine learning algorithms outperformed clinicians in terms of sensitivity and specificity
7	Machine learning approach for predicting underfive mortality determinants in Ethiopia: evidence from the 2016 Ethiopian demographic and health survey [27]	RF, LR, and KNN have been used. The highest accuracy is 67.2% with RF
8	Proposed stacking model	Stacking with the highest accuracy of 97.04%

## Data Availability

All the data related to this study will be provided upon request to the corresponding author.
